# Thiocarbamates from* Moringa oleifera* Seeds Bioactive against Virulent and Multidrug-Resistant* Vibrio* Species

**DOI:** 10.1155/2017/7963747

**Published:** 2017-07-09

**Authors:** Renata Albuquerque Costa, Oscarina Viana de Sousa, Ernesto Hofer, Jair Mafezoli, Francisco Geraldo Barbosa, Regine Helena Silva dos Fernandes Vieira

**Affiliations:** ^1^Federal University of Ceará, Fortaleza, CE, Brazil; ^2^Sea Sciences Institute, Federal University of Ceará, Fortaleza, CE, Brazil; ^3^Oswaldo Cruz Institute, Rio de Janeiro, RJ, Brazil; ^4^Department of Organic and Inorganic Chemistry, Federal University of Ceará, Fortaleza, CE, Brazil

## Abstract

Prospect of antibacterial agents may provide an alternative therapy for diseases caused by multidrug-resistant bacteria. This study aimed to evaluate the in vitro bioactivity of* Moringa oleifera* seed extracts against 100 vibrios isolated from the marine shrimp* Litopenaeus vannamei*. Ethanol extracts at low (MOS-E) and hot (MOS-ES) temperature are shown to be bioactive against 92% and 90% of the strains, respectively. The most efficient Minimum Inhibitory Concentration (MIC) levels of MOS-E and MOS-ES against a high percentage of strains were 32 *µ*g mL^−1^. Bioguided screening of bioactive compounds showed that the ethyl acetate fraction from both extracts was the only one that showed antibacterial activity. Vibriocidal substances, niazirine and niazimicine, were isolated from the aforementioned fraction through chromatographic fractionation.

## 1. Introduction

Bacteria of* Vibrio* genus are ubiquitous in the marine environment and are part of the indigenous microbiota of marine invertebrates. Some species are recognized as human pathogens, often associated with diseases such as cholera and acute gastroenteritis [1, 2]. Vibrios are also seen as opportunistic pathogens of cultured aquatic organisms, which is one of the reasons for observing the use of antibiotics in shrimp cultivation.

Furthermore, inappropriate use of antimicrobial drugs in aquaculture has been associated with negative environmental impacts: selection of bacterial populations resistant to drugs [3, 4] and contamination of adjacent ecosystems to culture ponds.

Thus, detection of antibacterial activity in higher plants against vibrios with virulent, antimicrobial-resistant profiles is of utmost importance. In the present research, due to its high medicinal activity [5], the vibriocidal capacity of the angiosperm* Moringa oleifera* was investigated. The antimicrobial effect of* Moringa* has been researched since the 1980s and seems to be related to some specific components including pterygospermin and* Moringa* glycosides, as well as 4-(*α*-L-rhamnosyloxy)-benzyl isothiocyanate and 4-(*α*-L-rhamnosyloxy)-phenyl-acetonitrile, which act especially against* Bacillus subtilis*,* Mycobacterium phlei*,* Serratia marcescens*,* Escherichia coli*,* Pseudomonas aeruginosa*,* Shigella* sp., and* Streptococcus* sp. [6].

Despite the extensive scientific evidence of the bioactivity of* Moringa* against bacteria [7–10], studies on its effects against vibrios are still incipient. Thus, this research aimed to evaluate the bioactive potential from extracts of* Moringa* seeds against vibrios with virulent and multidrug antimicrobial profile.

## 2. Material and Methods

### 2.1. Origin of the Strains

One hundred* Vibrio* sp. strains isolated from the hemolymph of* Litopenaeus vannamei* shrimp were used, all of which belonged to the bacteriological collection of the Laboratory of Environmental and Fishery Microbiology at Sea Sciences Institute (LABOMAR-UFC-Brazil). The 100 strains were phenotypically identified as* V. navarrensis* (*n* = 53),* V. brasiliensis* (*n* = 15),* V. parahaemolyticus* (*n* = 10),* V. xuii* (*n* = 8),* V. coralliilyticus* (*n* = 5),* V. cholerae* (*n* = 4),* V. neptunis* (*n* = 2),* V. alginolyticus* (*n* = 1),* V. diazotrophicus* (*n* = 1), and* V. vulnificus* B3 (*n* = 1) [11]. The enzymatic profile and antimicrobial resistance were used as a criterion for selection [12].

### 2.2. Botanical Material

*Moringa oleifera* seeds were collected from two specimens grown in a* campus* of the Federal University of Ceará (Pici, Fortaleza, Ceará). Separation from the fruit (pod), removal of husks, and posterior packing in plastic polyethylene bags followed the material collection.

### 2.3. *Moringa oleifera* Extracts

All extraction procedures were performed in the Department of Organic and Inorganic Chemistry at Federal University of Ceará (UFC). Part of the crushed seeds of* M. oleifera* (110 g) was subjected to three extractions with 300 mL cold hexane (PA) at 24 h intervals. After filtration and evaporation of the solvent under reduced pressure in a rotary evaporator, 15.36 g of an extract of fluid and yellowish appearance called MOS-H was obtained. The resulting cake was subjected to three cold extractions with 300 mL ethanol (PA) in 24 h intervals. After filtration and evaporation of the solvent under reduced pressure in a rotary evaporator, 11.64 g of an extract of fluid and dark appearance called MOS-E was obtained. 139 g of crushed* M. oleifera* seeds was used for hot extraction in a Soxhlet apparatus with 800 ml of hexane (PA) for 48 h. After filtration and evaporation of the solvent under reduced pressure in a rotary evaporator, 28.29 g of an extract of fluid and yellowish aspect called MOS-HS was obtained. Another extraction was carried out with 800 ml of ethanol (PA) for 48 h. After filtration and evaporation of the solvent under reduced pressure in a rotary evaporator, 13.66 g of a pasty and dark-colored appearance called MOS-ES was obtained.

### 2.4. In Vitro Susceptibility Testing of* Moringa oleifera* Extracts

Susceptibility of* Vibrio* sp. strains to the four extracts types (MOS-H, MOS-E, MOS-HS, and MOS-ES) was assessed using the disk diffusion method (DDM) and by Minimum Inhibitory Concentration (MIC) [13]. In order to proceed with the DDM, paper discs (6 mm) containing 100 *µ*L of each extract were applied in triplicate on Mueller-Hinton plates previously seeded with bacterial cultures (10^8^ UFC mL^−1^). As negative and positive Gram control, strains of* V. parahaemolyticus* IOC and* Staphylococcus aureus* ATCC 25923, respectively, were used. For MIC determination, macrodilution technique in Mueller-Hinton broth containing 1% NaCl was used. Concentrations of 4, 8, 16, 32, and 64 *µ*g mL^−1^ were tested, using the MOS-E oils (cold extraction with ethanol) and MOS-ES (hot extraction with ethanol) in comparison to isolates susceptible to crude extracts in the DDM test.

### 2.5. Chromatographic Fractionation of MOS-E and ES-MOS and Obtaining Active Fraction Ethyl Acetate (MOS-ESA)

Part of the MOS-E extract (3.1 g) was adsorbed onto 7.3 g of silica gel and chromatographed on 52.5 g of silica gel in open column (Ø 5,0 cm). Elution was done in order of increasing polarity with dichloromethane (1200 ml) (MOS-ED), ethyl acetate (800 mL) (MOS-EA), and methanol (600 mL) (MOS-EM). Solvents were evaporated under reduced pressure in a rotary evaporator, yielding the following mass and yields: MOS-ED 1,920.8 mg, 61.96%; MOS-EA 231.6 mg, 7.47%; MOS 660.8 mg, 21.31%. MOS-ES extract (2.6 g) was adsorbed onto 3.9 g of silica gel and chromatographed on 48.3 g of silica gel in open column (Ø 5,0 cm). Elution was done in order of increasing polarity with dichloromethane (500 ml) (MOS ESD), ethyl acetate (700 ml) (MOS-ESA), and methanol (600 ml) (MOS-ESM). Solvents were evaporated under reduced pressure in a rotary evaporator, yielding the following mass and yields: MOS-ED 34.8 mg, 1.33%; MOS-EA 335.3 mg, 12.89%; MOS 1,978 mg, 76.07%. All fractions were subjected to antimicrobial activity test by disk diffusion method. The bioactive fraction was subjected to chromatographic fractionation by High-Performance Liquid Chromatography (HPLC) in order to isolate its active principles.

### 2.6. Chromatographic Fractionation of the Ethyl Acetate Fraction (MOS-ESA) by High-Performance Liquid Chromatography (HPLC) and Isolation of Active Substances

**Figure 1:**
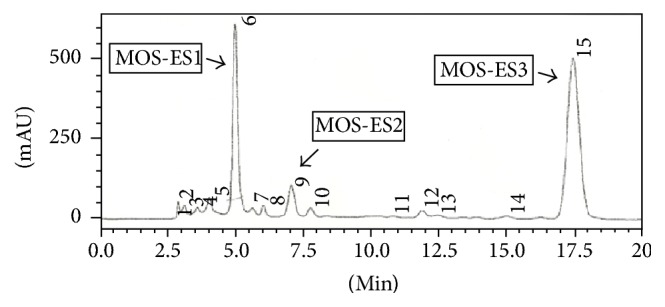
HPLC chromatogram analysis of an active fraction of MOS-ESA and isolation of MOS-ES-1, MOS-ES-2, and MOS-ES-3.

Part of the MOE-ESA active fraction (285 mg) was analyzed by HPLC in a chromatograph Shimadzu® (UFLC model) equipped with a UV-Vis detector with diode array (model SPD-M20A). Separation was performed in reverse phase conditions in semipreparative column (C-18.5 *μ*m), with isocratic elution using MeOH/H_2_O (1 : 1) with a 4.72 mL min^−1^ flow. Chromatographic fractionation of the ethyl acetate's fraction from the MOS-ES fixed oil resulted in the detection (at 284 nm) and isolation of three main substances (Figure 1), which were obtained as whitish, amorphous solids: the compound related to the peak 1 (23.3 m; *t*_*r*_ = 4.99 min) was called MOS-ES-1, related to peak 2 (4.0 mg; *t*_*r*_ = 7.06 min) was called MOS-ES-2, and related to peak 3 (65.1 mg, *t*_*r*_ = 17.45 min) was called MOS-ES-3. Isolated substances (S1 and S3) had their structures determined by the analysis of Nuclear Magnetic Resonance (NMR) and Infrared (IR) spectral data and also by comparison to other findings described in the literature [14, 15].

## 3. Results

### 3.1. Disk Diffusion Test

From all the 100 strains tested, only five were resistant to MOS-E fixed oil; on the other hand, 36 had its growth inhibited, with average inhibition zones ranging from 13 to 15 mm (Table 1). When comparing the oils, MOS-ES tests demonstrated inhibitory bacterial rate somewhat lower, since most strains (*n* = 37) had inhibitions indicated by halos in the range 10 to 12 mm (Table 2). In addition, seven strains were resistant to the MOS-ES. The larger inhibition halo (22 to 24 mm) was observed on MOS-E test against a* V. navarrensis* strain (Table 1). Both extracts showed antibacterial effect against the Gram-positive* (Staphylococcus aureus)* and Gram-negative* (Vibrio parahaemolyticus)* controls (Tables 1 and 2). MOS-H and MOS-HS extracts showed no bioactivity against any of the isolates (*n* = 100).

### 3.2. Minimum Inhibitory Concentration (MIC)

MIC levels of the MOS-E show that 83 (90.2%) of the strains were inhibited in the presence of a 32 *µ*g mL^−1^ concentration. Levels of 8, 16, and 64 *µ*g mL^−1^ were able to inhibit 1, 6, and 2 strains, respectively. Also, MIC levels of the MOS-ES able to inhibit the highest percentage of strains (*n* = 88; 97.8%) were that of 32 *µ*g mL^−1^. On the other hand, only 2 (2.2%) of the strains were inhibited by a MOS-ES MIC of 16 *µ*g mL.

### 3.3. Bioactivity of the Fractions

Study of the bioactivity of dichloromethane (CH_2_Cl_2_), ethyl acetate (EtOAc), and methanol (MeOH) fractions of MOS-E and MOS-ES revealed that only EA fractions from both extracts showed antimicrobial activity (Table 3). Ethyl acetate fractions were selected and subjected to chromatographic fractionation by HPLC in order to isolate bioactive substances.

### 3.4. Identification of Individual Substances

From the MOS-EA fraction, three substances were identified S1 (MOS-ES-1), S2 (MOS-ES-2), and S3 (MOS-ES-3). Due to the small amount obtained from MOS-ES-2 substance (S2), it was impossible to obtain spectroscopic data enough for a structural characterization. Those two substances derived from MOE-EA fraction were also obtained in small amounts and identified based only by Thin Layer Chromatography (TLC). Furthermore, the drain of this fraction yielded 23 mg of unidentified less polar substances.

### 3.5. Structural Characterization of MOS-ES-1 (S1) and MOS-ES-3 (S3)

S1 was isolated as a white amorphous solid and presented 129–132°C, [M + Na]^+^ = 302,1012. RMN ^1^H (500 MHz, CD_3_OD): *δ* 7,28 (2H, d,* J* = 8,6 Hz); 7,07 (2H, d,* J* = 8,6 Hz); 5,43 (1H, d,* J* = 1,4 Hz); 3,84 (1H, dd,* J* = 3,4 e 9,5 Hz); 3,82 (2H, s); 3,82 (2H, m); 3,62 (1H, m); 3,46 (1H, t,* J* = 9,5 Hz); 1,21 (3H, d,* J* = 6,2 Hz). RMN ^13^C (125 MHz, CD_3_OD): *δ* 157,4 (C-1); 118,1 (C-2 e C-6); 130,2 (C-3 e C-5); 125,8 (C-4); 22,7 (C-7); 119,8 (C-8); 99,9 (C-1′); 72,0 (C-2′); 72,0 (C-3′); 73,8 (C-4′); 70,7 (C-5′); 18,0 (C-6′). IV *ν*_max_/cm^−1^ (KBr) 3411 (*ν*_O-H_), 2948 (*ν*_-C-H_), 2250 (*ν*_C≡N_), 1611–1513 (*ν*_C=C_), 1227 (*ν*_C-O_).

Analysis of spectral data and the comparison with information in the literature [15] were used to characterize S1 as the 4-[(*α*-L-rhamnosyloxy)benzyl] nitrile or niazirine.

S3 was also isolated as a white amorphous solid and presented 130–133°C, [M + Cl]^−^ = 392,0951, RMN ^1^H (500 MHz, CD_3_OD): *δ* 7,25 (2H, d,* J* = 8,7 Hz); 7,01 (2H, d,* J* = 8,7 Hz); 5,40 (1H, d,* J* = 1,8 Hz); 4,63 (2H, s); 4,46 (2H, q,* J* = 7,1 Hz); 3,99 (1H, dd,* J* = 1,8 e 3,4 Hz); 3,83 (1H, dd,* J* = 3,4 e 9,5 Hz); 3,62 (1H, m); 3,45 (1H, t,* J* = 9,5 Hz); 1,29 (3H, t,* J* = 7,1 Hz); 1,21 (3H, d,* J* = 6,2 Hz), RMN ^13^C (125 MHz, CD_3_OD): *δ* 157,1 (C-1); 117,6 (C-2 e C-6); 130,1 (C-3 e C-5); 133,1 (C-4); 49,0 (C-7); 192,3 (C-8); 66,9 (C-9); 14,7 (C-10); 100,2 (C-1′); 72,1 (C-2′); 72,3 (C-3′); 73,9 (C-4′); 70,6 (C-5′); 18,0 (C-6′). IV *ν*_max_/cm^−1^ (KBr) 3405 (*ν*_O-H_), 2948 (*ν*_-C-H_), 1611–1513 (*ν*_C=C_), 1233 (*ν*_C=S_).

Analysis of spectral data and the comparison with information in the literature [14] were used to characterize S3 as* O*-ethyl-4-[(*α*-L-rhamnosyloxy)benzyl] thiocarbamate or niazimicine.

### 3.6. Evaluation of Antibacterial Activity of the Isolated Substances

Bioactivity results of S1 and S3 substances against ten* Vibrio* sp. strains are summarized in Table 4. It is possible to attest, in a comparison between substances 1 and 3 (S1 and S3) using the size of the inhibition zone as a criterion, a greater antibacterial efficiency of S3 against all strains.

## 4. Discussion

Studies on the bioactive properties of* Moringa* seeds highlight multiple uses of this phanerogam, for example, turbidity removal of contaminated water by coagulation [16], biosorption of heavy metals in effluents [17], anti-inflammatory [18], and antibacterial activity against* S. aureus* and* E. coli* [19]. However, bioactivity against vibrios has not been widely researched. Thus, the high inhibition level of both extracts (95% for MOS-E; 93% for MOS-ES) (Tables 1 and 2) against antimicrobial-resistant vibrios with virulence factors must be stressed. This vibriocidal activity is congruent with the findings of Vieira et al. (2010), who investigated antibacterial activity of ethanol extracts of* Moringa* seeds and found inhibition zones ranging from 26 to 29.5 mm against classical* V. cholerae* 569B.

Vibriocidal activity of aerial parts of* M. oleifera* was reported by Peixoto et al. [20]. The authors tested the bioactivity of its extracts against standard* V. parahaemolyticus* strain and found average inhibition halos of 21.9 and 20.7 mm for ethanol and aqueous extracts, respectively.

Moringa seed extracts have also been used in tests against standard strain of* V. cholerae*. Atieno et al. [21] observed the bioactivity of hexane and methanolic extracts of* M. oleifera* and* M. stenopetala* seeds against* Salmonella* ser. Typhi,* E. coli*, and* V. cholerae.* For the species of* Vibrio*, the authors reported inhibition halo sizes of 22.2 and 13.8 mm for hexane and methanolic extracts of* M. oleifera*, respectively. The aforementioned data support the assertion that* Moringa* seeds have vibriocidal potential; however, it cannot be compared to the ones presented in the present study, since antibacterial activity in leaf extracts was not detected (MOS-H and MOS-HS).

Despite the occurrence of compounds with antibacterial activity in different parts of* Moringa* is being reported in the scientific literature since the early 1980s [22], their use for epizootic purposes has been little explored. The data obtained by the disk diffusion test and the results of the Minimum Inhibitory Concentration (MIC) serve as evidence of the high antibacterial potential of ethanol extracts of MOS-E and MOS-ES against vibrios.

Satisfactory results in disk diffusion tests and the definition of MIC levels were pivotal for the decision of carrying out complementary studies, starting with the bioguided screening of bioactive compounds. The selective antimicrobial effect of* Moringa*'s crude extract fractions was also verified by Nantachit [23]. The author noted that the dichloromethane fraction was active against* E. coli*.

Guevara et al. [24] reported similar structures described in this study, namely,* O*-ethyl-4-(*α*-L-rhamnosyloxy) benzyl carbamates, 4(*α*-L-rhamnosyloxy)-benzyl isothiocyanate, niazimicine, 3-*O*-(6′-*O*-oleoyl-*β*-D-glucopyranosyl)-*β*-sitosterol, *β*-sitosterol-3-*O*-*β*-D-glucopyranoside, niazirine, *β*-sitosterol, and glycerol-1-(9-octadecanoate) by studying the ethanol extracts of* Moringa* seeds. The authors mentioned the bioactivity potential of niazimicine and niazirine. Furthermore, Gilani et al. [25] isolated four bioactive compounds out of the bioguided fractionation of ethanolic extracts of* M. oleifera* leaves and also found compatible substances with those described in the present study: niazinine A, niazinine B niazinine, niazimicine, and niazinine A + B.

In this context, Padla et al. [10] isolated 4-(alpha-L-Rhamnosyloxy)benzyl isothiocyanate and 4-(4′-O-acetyl-alpha-L-rhamnosyloxy)-benzyl isothiocyanate from* Moringa oleifera* seeds and demonstrated that both substances were bioactive against Gram-positive bacteria* Staphylococcus aureus*,* Staphylococcus epidermidis*, and* Bacillus subtilis*. In the present study, we isolated from* Moringa* seeds the substances (1) 4-[(*α*-L-rhamnosyloxy)benzyl]nitrile (niazirine) and (2)* O*-ethyl-4-[(*α*-L-rhamnosyloxy)benzyl] thiocarbamate (niazimicine).

The compounds* O*-ethyl-*p*-hydroxybenzene carbamate and* O*-methyl-4-[(2′,3′,4′-tri-*O*-acetyl-*α*-L-rhamnosyloxy)benzyl] thiocarbamate must also be added to the previously mentioned substances as isolates from leaf extracts of* M. oleifera* [26, 27]. The addition of methanol or ethanol to isothiocyanate is considered a pathway to the synthesis of thiocarbamates glycosides in* Moringa* [26].

Since the 1990s, biological activities of pharmacological interest concerning nitriles isolated from* Moringa* have been described, and its antitumor [28] and antihypertensive [14] functions are noteworthy, along its use in the prevention of carcinogenesis [24].

The presence of niazimicine in* Moringa* seeds is often cited as a strong antitumor factor. It can be used as a prophylactic or therapeutic measure while treating HSV-1 infections [29].

Although this is not the first description of carbamate glycosides in constituent parts of the* M. oleifera* species, its unexploited vibriocidal potential must be highlighted. In the present study, niazirine and niazimicine showed high antibacterial efficiency against vibrios with phenotypic profiles compatible to the presence of virulence factors (exoenzymes and *β*-hemolysis producers), cross-resistance to *β*-lactams, and mono/multiresistance to antibiotics. These findings suggest a new class vibriocidal compounds. Moreover, the results are consistent with the demand for new alternatives to antibacterial drugs in order to mitigate the impact caused by the indiscriminate use of antimicrobials in aquaculture.

## Figures and Tables

**Table 1:** Distribution, according to the average inhibition zone (mm), of the number of strains inhibited by cold ethanol extract of *Moringa oleifera* seeds (MOS-E).

*Vibrio* species	*n*	Average inhibition zone (mm)						
		22–24	19–21	16–18	13–15	10–12	7–9	0
*V. navarrensis*	53	1	2	5	27	15	3	—
*V. brasiliensis*	15	—	1	2	4	2	5	1
*V. parahaemolyticus*	10	—	—	—	—	—	9	1
*V. xuii*	8	—	—	—	1	—	5	2
*V. coralliilyticus*	5	—	—	2	1	1	1	—
*V. cholerae*	4	—	—	—	2	1	1	—
*V. neptunis*	2	—	—	1	—	1	—	—
*V. alginolyticus*	1	—	—	—	1	—	—	—
*V. diazotrophicus*	1	—	1	—	—	—	—	—
*V. vulnificus *B3	1	—	—	—	—	—	—	1

*V. parahaemolyticus* IOC^a^	1	—	—	1	—	—	—	—
*S. aureus* ATCC25923^b^	1	—	1	—	—	—	—	—

Total	102	1	5	11	36	20	24	5

*n*: number of isolates. a: standard strain used as Gram-negative control. b: standard strain used as Gram-positive control.

**Table 2:** Distribution, according to the average inhibition zone (mm), of the number of strains inhibited by hot ethanol extract of *Moringa oleifera* (MOS-ES).

*Vibrio* species	*n*	Average inhibition zone (mm)					
		19–21	16–18	13–15	10–12	7–9	0
*V. navarrensis*	53	1	6	14	26	5	1
*V. brasiliensis*	15	1	2	2	4	3	3
*V. parahaemolyticus*	10	—	—	—	—	9	1
*V. xuii*	8	—	—	—	1	7	—
*V. coralliilyticus*	5	—	—	1	3	1	—
*V. cholerae*	4	—	—	1	1	1	1
*V. neptunis*	2	—	—	—	2	—	—
*V. alginolyticus*	1	—	—	1	—	—	—
*V. diazotrophicus*	1	—	1	—	—	—	—
*V. vulnificus *B3	1	—	—	—	—	—	1

*V. parahaemolyticus* IOC^a^	1	—	1	—	—	—	—
*S. aureus *ATCC25923^b^	1	—	1	—	—	—	—

Total	102	2	11	19	37	26	7

*n*: number of isolates. a: standard strain used as Gram-negative control. b: standard strain used as Gram-positive control.

**Table 3:** Average inhibition halos of ethyl acetate (EtOAc) fractions of ethanolic extracts of *Moringa oleifera* seeds extracted cold (MOS-E) and hot (MOS-ES) with ethanol against ten *Vibrio* sp. strains isolated from the hemolymph of *Litopenaeus vannamei*.

Strain	*Vibrio *species	EtOAc (MOS-E)	EtOAc (MOS-ES)
1	*V. coralliilyticus*	14.51 ± 0.07	12.10 ± 0.08
7	*V. alginolyticus*	12.65 ± 0.06	10.52 ± 0.45
13	*V. navarrensis*	15.71 ± 0.21	14.03 ± 0.07
35	*V. diazotrophicus*	17.14 ± 0.04	16.58 ± 0.42
40	*V. navarrensis*	15.75 ± 0.35	11.82 ± 0.31
42	*V. xuii*	12.39 ± 0.24	11.80 ± 0.25
46	*V. parahaemolyticus*	8.53 ± 0.15	7.66 ± 0.12
89	*V. neptunis*	18.54 ± 0.42	11.18 ± 0.09
97	*V. cholerae*	8.46 ± 0.09	7.32 ± 0.10
98	*V. brasiliensis*	15.01 ± 0.05	14.61 ± 0.15

**Table 4:** Average size of inhibition halos from substances 1 (S1) and 3 (S3) isolated from fractions of ethyl acetate (EtOAc) of *Moringa* seed in cold extraction with ethanol (MOS-E) and in hot extraction with ethanol (MOS-ES), against ten *Vibrio* sp. strains isolated from the hemolymph of *Litopenaeus vannamei*.

Strains	Species	Fraction of EtOAc MOS-ES		
		S1	S2	S3
1	*V. coralliilyticus*	8.11 ± 0.09	nt	14.04 ± 0.02
7	*V. alginolyticus*	8.32 ± 0.02	nt	15.98 ± 0.09
13	*V. navarrensis*	9.76 ± 0.43	nt	19.89 ± 0.11
35	*V. diazotrophicus*	7.42 ± 0.24	nt	21.21 ± 0.24
40	*V. navarrensis*	9.77 ± 0.15	nt	17.89 ± 0.12
42	*V. xuii*	8.76 ± 0.29	nt	20.33 ± 0.06
46	*V. parahaemolyticus*	7.66 ± 0.45	nt	9.90 ± 0.07
89	*V. neptunis*	7.14 ± 0.11	nt	20.11 ± 0.03
97	*V. cholerae*	7.30 ± 0.27	nt	10.15 ± 0.18
98	*V. brasiliensis*	7.99 ± 0.02	nt	18.75 ± 0.06

nt: not tested.
